# A new understanding of radiographic landmarks of the greater trochanter that indicate correct femoral rotation for measurement of femoral offset

**DOI:** 10.1186/s42836-022-00121-y

**Published:** 2022-06-01

**Authors:** Jakub Tatka, Dimitri Delagrammaticas, Bryson R. Kemler, Samuel I. Rosenberg, Alex W. Brady, Anna R. Bryniarski, Grant J. Dornan, Joel M. Matta

**Affiliations:** 1 The Steadman Clinic, Vail, CO USA; 2 Steadman Philippon Research Institute, 181 W Meadow Drive, Suite 1000, Vail, CO 81657 USA

**Keywords:** Hip arthroplasty, Femoral offset, Femoral rotation, Greater trochanter

## Abstract

**Objectives:**

To establish and validate a novel method for aligning femoral rotation to accurately measure femoral offset for preoperative templating and component sizing, and to identify the physical location of two radiographic lines utilized in the described method.

**Materials and methods:**

Cadaveric proximal femurs were skeletonized and mounted to a biaxial load frame. Two radiographic lines along the greater trochanter were identified fluoroscopically. The femurs were rotated, and images were taken when the lines appeared superimposed, then in 2-degree increments to 10° of internal and external rotation, and at 30°. Radiographic femoral offset was calculated at each angle, and the maximum and aligned offsets were compared. Bone was removed until the radiographic lines disappeared, then a metal wire was inserted in place of the bone to confirm that the lines reappeared.

**Results:**

The physical locations of the radiographic landmarks were on the anterior and posterior aspects of the greater trochanter. The mean true femoral offset was 38.2 mm (range, 30.5–46.3 mm). The mean aligned femoral offset was 37.3 mm (range, 29.3–46.3 mm), a 2.4% underestimation. The mean angle between aligned and true offset was 3.6° of external rotation (range, 10°ER-8°IR). Intra-rater intraclass correlation coefficient was 0.991.

**Conclusion:**

Alignment of the radiographic lines created by the anterior and posterior aspects of the greater trochanter is a reliable and accurate rotational positioning method for measuring true femoral offset when using plain films or fluoroscopy, which can aid surgeons with preoperative templating and intraoperative component placement for total hip arthroplasty.

## Introduction

Total hip arthroplasty (THA) is performed on over 370,000 hips per year in the United States [1]. Restoration of native hip anatomy and biomechanics, such as center or rotation, leg length, and femoral offset, is integral for restoring function. Preoperative templating via plain radiography is a standard practice to determine prosthetic size and position based on a patient’s native femoral offset, which is defined as the perpendicular distance from the center of rotation of the femoral head to the long axis of the proximal femoral shaft [2]. Subsequent prosthetic reproduction of femoral offset in THA is important for achieving maximum abductor force and active range of motion and preventing impingement and dislocation [3–6], and the need to accurately measure femoral offset on plain radiograph has become increasingly recognized in recent years [7].

**Fig. 1:**
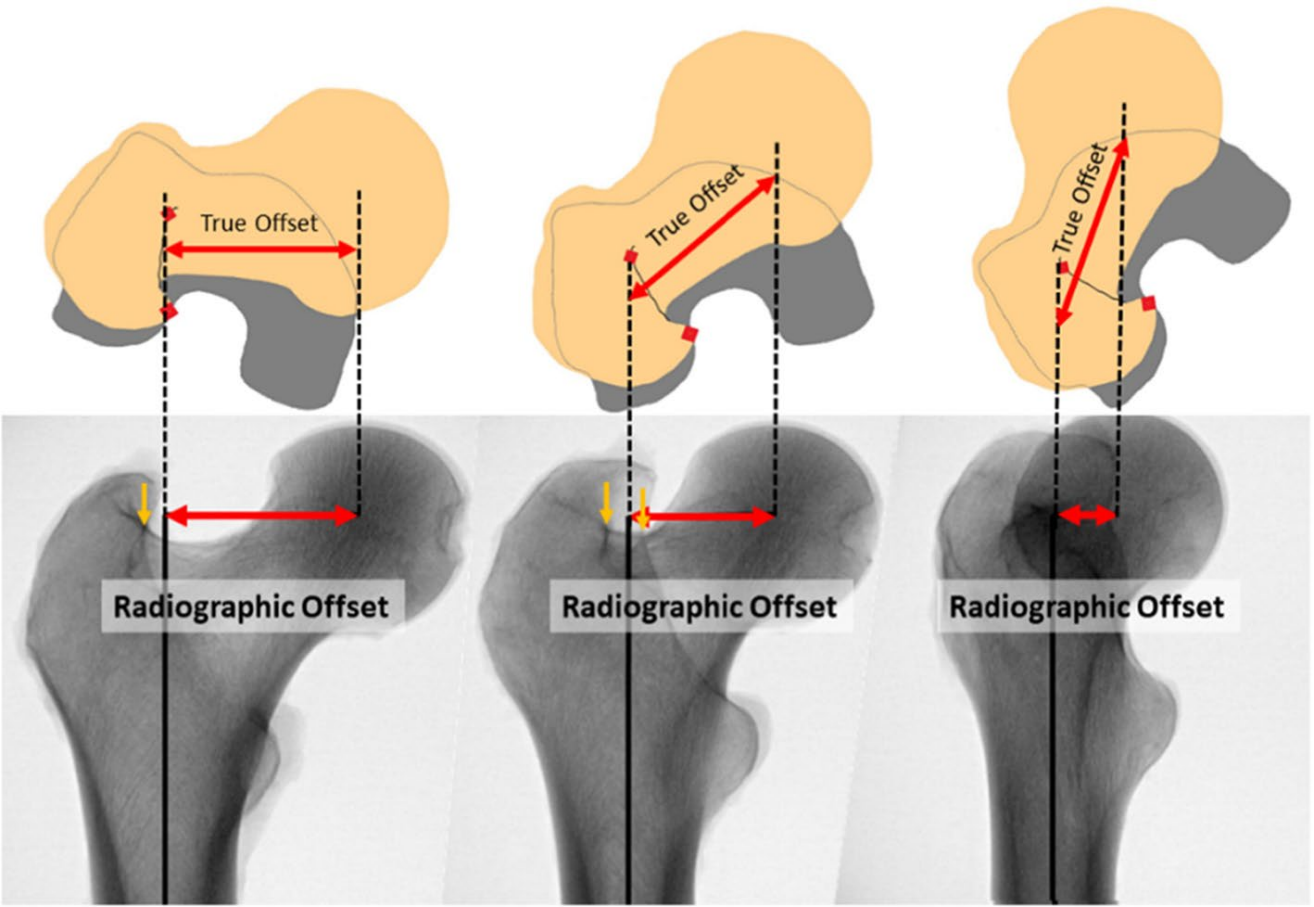
Three radiographic images of proximal femurs at different degrees of external rotation, with a schematic view of their orientation in the transverse plane. The offset measured on the radiographs diminishes as the angle of external rotation increases, while the true offset remains the same. The vertical yellow arrows indicate the position of the radiographic lines, which are not visible on the right-hand image because the rotation is too extreme. The two red squares on the schematic images represent the physical location of the radiographic lines. The left-hand image demonstrates our hypothesis that the true offset can be measured radiographically when the radiographic lines appear superimposed

When using plain films to measure femoral offset, the X-ray beam must be perpendicular to the plane determined by the intersection of the center lines of the neck and shaft. All other degrees of rotation will show less-than-true-value offset (Fig. 1). Anteroposterior radiographs of the pelvis may underestimate femoral offset by as much as 13%, yet this view remains standard when templating for THA [2]. Despite the long history and high frequency of THA, and the associated millions of radiographic measurements, there is plenty of qualitative guidance to internally rotate the extremity and to minimize the appearance of the lesser trochanter on the image exists, but very limited quantitative guidance is available [7, 8].

While performing THA with fluoroscopy, the senior author observed that two vertical radiographic lines created by the medial portion of the greater trochanter were superimposed during internal rotation of the femur. It was also observed that when these two lines were superimposed, femoral offset appeared to be at a maximum, representing the true offset.

While the posterior line results from the most posterior cortex of the medial greater trochanter, the physical anatomic location correlating with the anterior radiographic line remains unknown. Thus, the purpose of this study was to propose a method for aligning femoral rotation to accurately measure femoral offset for preoperative templating and component sizing, and to identify the physical location of two radiographic lines utilized in the described method. It was hypothesized that the anterior line was created by the convex arc of cortical bone within the fossa of the posterior greater trochanter and that the position for measuring true femoral offset is closely approximated by aligning these two radiographic lines.

## Materials and methods

### Specimen preparation

Eleven unpaired fresh-frozen cadaveric male and female hip specimens (mean age: 71.5, with range of 43–89; 5 males/6 females, 5 right /6 left) were used in this study. All specimens were dissected free of skin, soft tissues, and muscles to the level of the hip capsule, and then the joint was disarticulated. The proximal femur was then dissected of all soft tissues at the proximal shaft, femoral neck, and intertrochanteric line, leaving only the bone and cartilage intact. The femur was cut 15 cm distal to the lesser trochanter and the distal end was potted in polymethylmethacrylate (PMMA, Fricke Dental, Streamwood, IL) in a cylindrical mold with the long axis of the femur along the central long axis of the cylindrical mold.

### Testing

The proximal femur was mounted to a custom-designed fixture attached to the actuator of the dynamic tensile testing machine (ElectroPuls E10000, Instron Corp., Norwood, MA, USA). (Fig. 3). The rigid fixation of the femur in the actuator allowed for precise rotation of the proximal femur along its long axis. A fluoroscope was then directed perpendicular to the long axis of the femur. Neutral position of the femur was defined as the angle at which the anterior and posterior aspects of the greater trochanter were radiographically aligned. This was chosen as the reference from which to measure rotation, as it was hypothesized that this position closely approximates the position of maximum femoral offset. A radiograph was recorded at neutral, and at 2° increments from neutral to 10° of internal rotation and 10° of external rotation. Radiographs were also taken at 30° of internal and 30° of external rotation; 15 total radiographs were recorded for each specimen (− 30, − 10, − 8, − 6, − 4, − 2, 0, + 2, + 4, + 6, + 8, + 10, + 30). A metal calibration sphere with a 1-inch diameter was placed in the plane of the femur to calculate the pixel to mm scale.

**Fig. 2:**
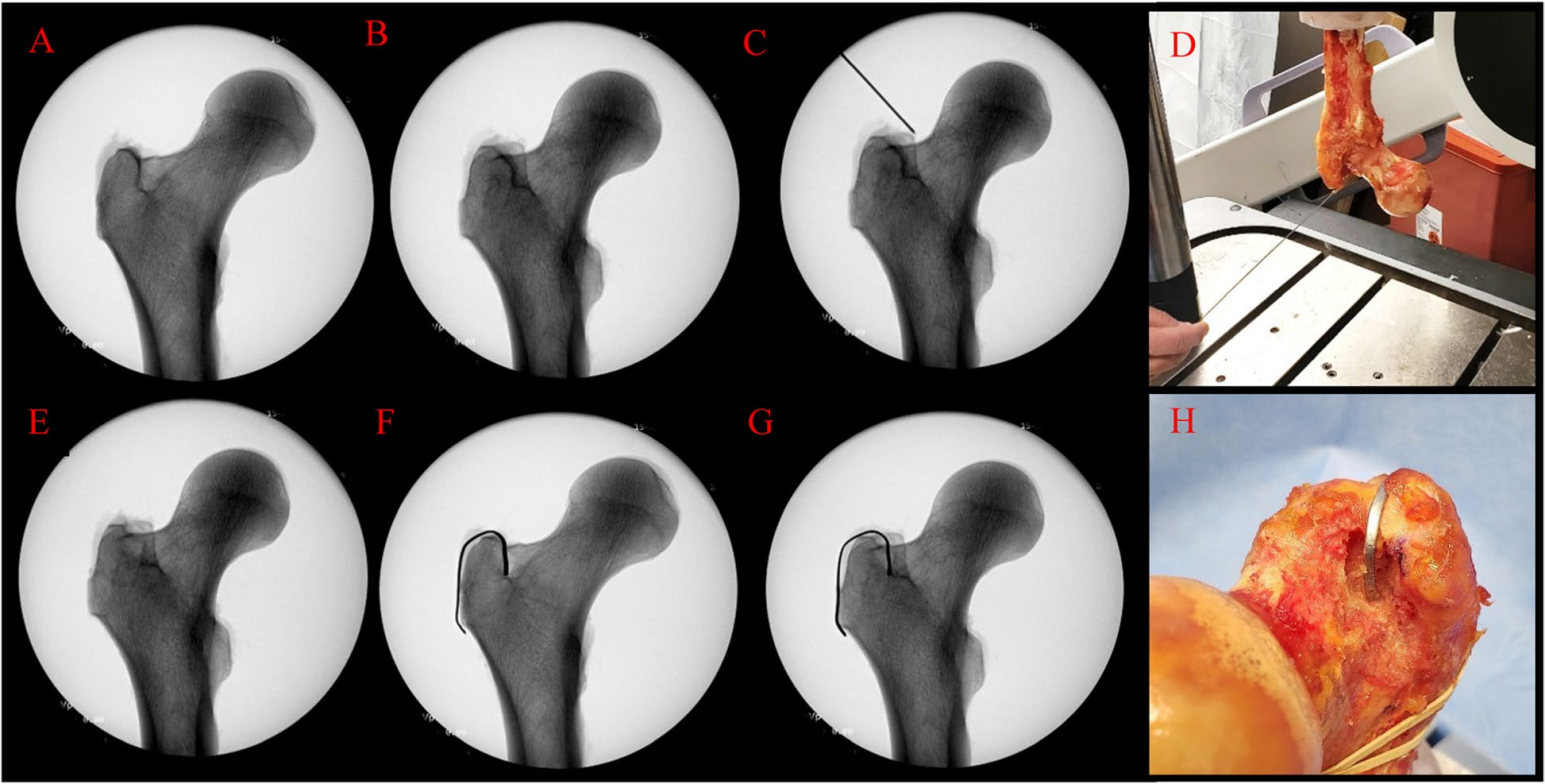
Radiographic and photographic images demonstrating (**A**) the presence of two superimposed radiographic lines on the medial greater trochanter at 0 degrees, (**B**) the presence of two radiographic lines on the medial greater trochanter at 30 degrees, (**C**) a K-wire in line with the posterior line radiographically, (**D**) the physical location of the K-wire as the radiograph in image c was taken, (**E**) the disappearance of the anterior radiographic line after bone has been removed at 30 degrees, (**F**) the reappearance of the anterior greater trochanteric line when the lead strip is placed in the center of the removed bone at 0 degrees, (**G**) the reappearance of the anterior greater trochanteric line when the lead strip is placed in the center of the removed bone at 30 degrees, (**H**) the physical location of the lead strip corresponding to the reappearance of the radiographic lines in images **f** and **g**

One femur was utilized to identify the physical location of the radiographic lines. Lead wire strips were placed on the cortex in the suspected region of the anterior radiographic lines. X-ray images were taken at two precise locations, neutral and 30 degrees of internal rotation, to confirm that this was the area of interest. Bone was removed from the anterior greater trochanteric fossa starting at the area beneath the lead strip and an X-ray image was taken again at neutral and 30 degrees of internal rotation to determine if the radiographic lines disappeared. Bone was removed incrementally, and images were taken until the radiographic lines disappeared. Lead strips were then substituted for the removed cortex, which demonstrated a return of the radiographic lines in the proper alignment (Fig. 2).

### Femoral offset calculation

**Fig. 3:**
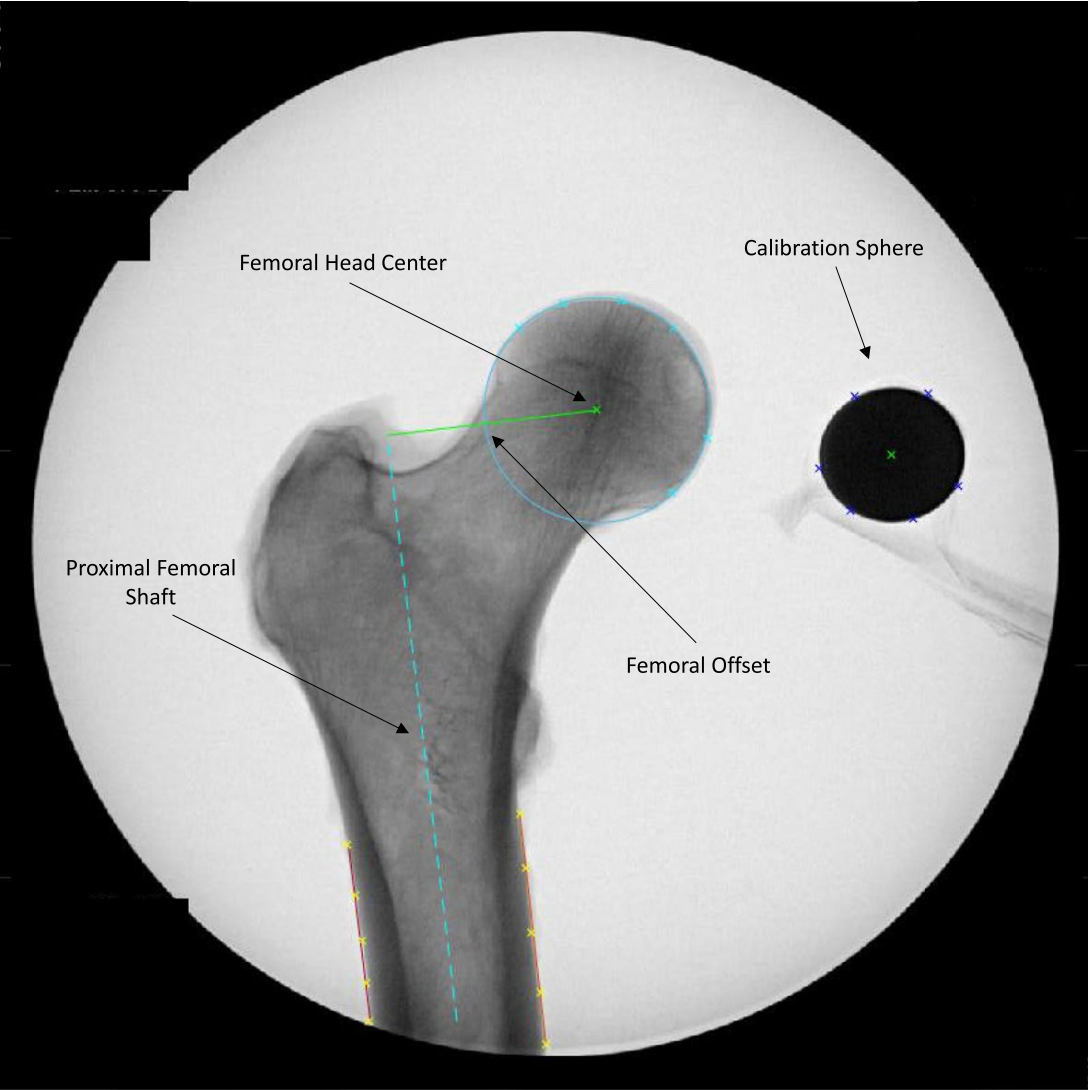
Image of femoral offset calculation from the custom software. The small crosses represent the points collected by the rater to perform the measurement. The blue dashed line represents the axis femoral shaft, calculated as the bisector of the orange and purple lines along the cortices of the femoral shaft. The green line represents the femoral offset

Using a custom software script written in MATLAB (Version 2014b, MathWorks, Natick, MA), femoral offset was calculated by defining the proximal femoral axis and measuring the perpendicular distance from this axis to the center of the femoral head. The proximal femoral axis was calculated by creating best-fit lines of 5 points on both the medial and lateral borders of the outer cortex, and then extrapolating the bisector of those lines. The center of the femoral head was calculated by marking 6 points around the spherical femoral head and then creating a best-fit circle from these points. A best-fit circle was also calculated in the same manner about the 1-inch metal calibration sphere to calculate the pixel to mm conversion ratio (Fig. 3). Measurements were taken 3 times by a single rater at each femoral rotation, with a one-week interval between measurements. The rater was blinded to the rotation of the specimen on the images, and the order of the images was randomized within each specimen. The femoral offset values were analyzed for intrarater reliability, as described in the statistical analysis section. True offset was defined as the maximum offset of all positions measured.

### Statistical analysis

Descriptive analysis was performed to assess the relationship between rotation angle and femoral offset. To assess intrarater measurement repeatability of the femoral offset measurements, three rounds of measurement were performed by a single rater. A two-way random effects model was used to calculate the single measures, absolute agreement version of the intraclass correlation coefficient (ICC). Non-parametric 95% bootstrap confidence intervals were reported. The ICC values were interpreted as follows: ICC < 0.40 = poor agreement; 0.4 < ICC < 0.75 = fair to good agreement; ICC > 0.75 = excellent agreement [9]. All statistical analyses were performed with the statistical package R, version 3.5.2 (R Development Core Team, Vienna, Austria) [10–12].

External rotation angles were reported as negative values while internal rotation angles were reported as positive values.

## Results

### Anatomic location of radiographic lines

The posterior line was confirmed to be produced by the most posterior aspect of the medial greater trochanter. The anterior radiographic line was created by the most anterior portion of the fossa of the posterior greater trochanter.

### Femoral offset

The mean true femoral offset was observed to be 38.2 mm (SD 4.9 mm, median 37.8 mm, range 30.5 to 46.3 mm), while the mean femoral offset measured at the radiographically aligned position was 37.3 mm (SD 5.4 mm, median 37.2 mm, range 29.8 to 46.3 mm). The mean underestimation error (the difference between calculated offset at neutral rotation and observed maximum femoral offset) was 0.9 mm, or 2.4% (median, 0.7; range, 0 to 2.1 mm). The mean range between highest and lowest femoral offset measured between − 10 and + 10° of rotation was 2.7 mm (median 2.7 mm, range 1.3 to 4.7 mm).

**Fig. 4:**
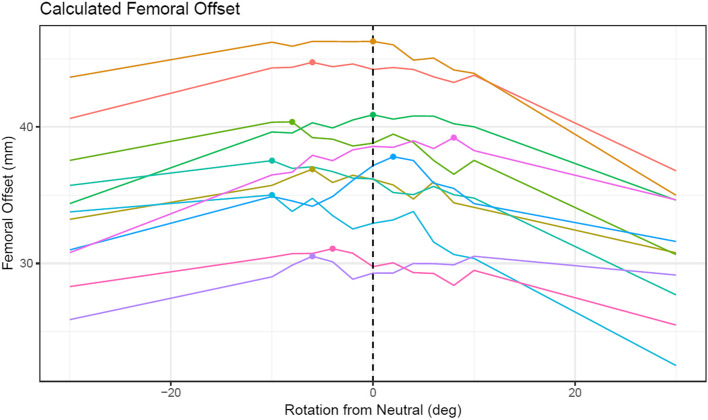
Graph of each specimen’s radiographic femoral offset in mm as a function of rotation angle from neutral rotation in degrees, where neutral corresponds to alignment of the radiographic trochanteric lines. Negative rotation represents external rotation while positive rotation represents internal rotation. Dots denote the maximum offset for each specimen

The mean rotation between the true offset position and the radiographically aligned position was − 3.6° (SD 5.6, median − 6°, range − 10° to + 8°). Two specimens had their maximum offset at 0 degrees. Maximum femoral offset was found at − 10° for two specimens, − 8° for one specimen, − 6° for three specimens, − 4° for one specimen, + 2° for one specimen, and + 8° for one specimen. A plot of all specimens’ calculated offsets at each degree of rotation is presented in Fig. 4.

### Intrarater reliability

Intrarater reliability was excellent among the three rounds of measurement (Agreement ICC = 0.991, bootstrap 95% CI [0.988, 0.994]).

## Discussion

The present study describes a reproducible method to rotationally position the femur and to measure true femoral offset from plain radiographic images with an mean error of less than 1 mm. Prior research has consistently demonstrated that current radiographic methods of assessing femoral offset are not dependable.

Preoperative templating for THA is performed via AP pelvic X-rays taken on a supine patient with their hip internally rotated 10–15 degrees, which accounts for femoral anteversion and more closely approximates true femoral offset than a neutral or externally rotated hip [8]. However, Pasquier et al. demonstrated that radiographic methods underestimated femoral offset, compared to CT, by 8%, with a mean difference of 3.2 mm [13]. Sariali et al. showed a similar difference, with AP radiography underestimating femoral offset by 3.5 mm [14]. In a cadaveric study performed by Weber et al, 35% of radiographic femoral offset measurements exceeded a 5 mm difference from CT measurements, while leg length and global offset were more reliably within this range, at 1% and 15% respectively [15]. Blumel et al. reported findings that showed a projection error greater than 10% when the distance between radiographic double lines exceeded 5 mm [7]. This consistent underestimation of femoral offset can exert significant effects on patient outcomes following THA, as decreased postoperative femoral offset negatively affects muscle tensioning, joint stability, implant wear, and leg length perception [3–6, 16]. It has been shown that abductor strength following THA was best when femoral offset was maintained or increased slightly, and that functional deficits in abductor strength began to appear when femoral offset is decreased by 12%, or 5 mm [17]. Thus, the consistent underestimation of femoral offset by current methods provides little buffer to avoid these functional deficits. While CT templating has been proposed for its increased accuracy [13, 18], templating via standardized AP hip or pelvis radiography remains the accepted practice due to its availability, affordability and reduced radiation exposure. The method described in this study is significant in its potential to improve radiographic preoperative templating and patient outcomes in THA.

Additionally, the present study is one of the first to provide a radioanatomic description of the posterior contours of the medial aspect of the greater trochanter, defining the relationship between the physical anatomy and the corresponding radiodensities seen on two-dimensional imaging. Of note, the anterior radiographic line is produced by the convex arc of the fossa of the greater trochanter, a surface observed to be approximately perpendicular to the true femoral offset. While a future study with CT validation is necessary to prove this claim, this observation serves as one possible reason why the alignment of these radiographic lines produces the femoral rotation for accurate measurement of femoral offset. In addition to its utility in determining femoral offset for THA preoperative templating of any approach, these radioanatomic landmarks are readily identifiable in a supine patient allowing for intraoperative use of fluoroscopy with an anterior approach. Intraoperative use of fluoroscopy in THA has been shown to improve acetabular component placement [19] and reduce limb length discrepancy [20].

The authors acknowledge several limitations to this study. First, the study was performed on eleven cadaveric specimens, and a larger sample size may better represent the general population. However, Audigé et al. determined a minimum sample size of ten subjects is sufficient for analyzing a new measurement method [21]. Additionally, repeated measurements and intrarater correlation coefficient were used to increase the reliability of these results. Two specimens had maximum offsets at 10 degrees of ER, therefore the true offset could have been outside the measured range on those specimens. The use of isolated femora ensured unrestricted femoral rotation, but clinical use of this technique may be limited by a patient’s range of motion. Patients with severe osteoarthritis commonly have external rotation contracture [2, 22], which may interfere with the femoral rotation needed to align these trochanteric lines on imaging. Pain and body habitus may also prevent use of this method on the arthritic hip. However, these limitations of rotation can be addressed by measuring a healthy, unrestricted contralateral hip or altering the direction of the X-ray beam to show true offset. In any case, non-alignment of the greater trochanteric lines indicates a projection of less than true offset.

## Conclusion

To our knowledge, this study presents one of the first quantifiable, reliable, and reproducible method for calculation of maximum offset using two-dimensional imaging. This method can be used in clinical practice by surgeons to accurately identify maximum offset on preoperative templating with X-ray and intraoperative assessment via fluoroscopy, which will aid in THA implant placement, ball-head length, and offset sizing and improve patient outcomes.

## Data Availability

The datasets generated or analyzed during the current study are available from the corresponding author on reasonable request.

## Abbreviations

THA: Total hip arthroplasty
ICC: Intraclass correlation coefficient
SD: Standard deviation
mm: Millimeter
CI: Confidence interval
AP: Anteroposterior
CT: Computed tomography
ER: External rotation
IR: Internal rotation
